# miR-148b-3p inhibits gastric cancer metastasis by inhibiting the Dock6/Rac1/Cdc42 axis

**DOI:** 10.1186/s13046-018-0729-z

**Published:** 2018-03-27

**Authors:** Xiaowei Li, Mingzuo Jiang, Di Chen, Bing Xu, Rui Wang, Yi Chu, Weijie Wang, Lin Zhou, Zhijie Lei, Yongzhan Nie, Daiming Fan, Yulong Shang, Kaichun Wu, Jie Liang

**Affiliations:** 1 State Key Laboratory of Cancer Biology & National Clinical Research Center for Digestive Diseases and Xijing Hospital of Digestive Diseases, Fourth Military Medical University, 127 West Changle Road, Xi’an, Shaanxi 710032 China; 2 Department of Gastroenterology, Second Affiliated Hospital of Xi’an Jiaotong University, Xi’an, Shaanxi Province 710004 China; 3 National-Local Joint Engineering Research Center of Biodiagnostics & Biotheraphy, the Second Affiliated Hospital of Xi’an Jiaotong University, Xi’an, Shaanxi 710032 China; 4 Department of Gastroenterology, Nanjing Drum Tower Hospital, The Affiliated Hospital of Nanjing University Medical School, Nanjing, 210008 China

**Keywords:** Rho GTPase, GEFs, Dock6, Gastric cancer, Metastasis

## Abstract

**Background:**

Our previous work showed that some Rho GTPases, including Rho, Rac1 and Cdc42, play critical roles in gastric cancer (GC); however, how they are regulated in GC remains largely unknown. In this study, we aimed to investigate the roles and molecular mechanisms of Dock6, an atypical Rho guanine nucleotide exchange factor (GEF), in GC metastasis.

**Methods:**

The expression levels of Dock6 and miR-148b-3p in GC tissues and paired nontumor tissues were determined by immunohistochemistry (IHC) and in situ hybridization (ISH), respectively. The correlation between Dock6/miR-148b-3p expression and the overall survival of GC patients was calculated by the Kaplan-Meier method and log-rank test. The roles of Dock6 and miR-148b-3p in GC were investigated by in vitro and in vivo functional studies. Rac1 and Cdc42 activation was investigated by GST pull-down assays. The inhibition of Dock6 transcription by miR-148b-3p was determined by luciferase reporter assays.

**Results:**

A significant increase in Dock6 expression was found in GC tissues compared with nontumor tissues, and its positive expression was associated with lymph node metastasis and a higher TNM stage. Patients with positive Dock6 expression exhibited shorter overall survival periods than patients with negative Dock6 expression. Dock6 promoted GC migration and invasion by increasing the activation of Rac1 and Cdc42. miR-148b-3p expression was negatively correlated with Dock6 expression in GC, and it decreased the motility of GC cells by inhibiting the Dock6/Rac1/Cdc42 axis.

**Conclusions:**

Dock6 was over-expressed in GC tissues, and its positive expression was associated with GC metastasis and indicated poor prognosis of GC patients. Targeting of Dock6 by miR-148b-3p could activate Rac1 and Cdc42, directly affecting the motility of GC cells. Targeting the Dock6-Rac1/Cdc42 axis could serve as a new therapeutic strategy for GC treatment.

**Electronic supplementary material:**

The online version of this article (10.1186/s13046-018-0729-z) contains supplementary material, which is available to authorized users.

## Background

Gastric cancer (GC) is one of the most common malignancies and a leading cause of cancer-related deaths worldwide [1, 2]. Most GC-associated deaths can be attributed to cancer recurrence and metastasis, but the underlying mechanisms remain largely unknown [3, 4].

In humans, the Rho GTPase family comprises 20 Rho small G-proteins that can be categorized into the Cdc42 subgroup (Cdc42, RhoJ and RhoQ), the Rac subgroup (Rac1, Rac2, Rac3 and RhoG), the Rho subgroup (RhoA, RhoB and RhoC) and other less characterized subgroups [5, 6]. They play critical roles in cell proliferation, cell motility, tumor cell malignant transformation, and cancer metastasis and invasion [7]. Rho GTPases have a GTP-bound active state and a GDP-bound inactive state [8]. Rho GTPase activating proteins (GAPs) inactivate Rho GTPases by stimulating the hydrolysis of GTP, guanine nucleotide exchange factors (GEFs) “turn on” Rho GTPases by promoting the exchange of GDP for GTP, and Rho guanine nucleotide dissociation inhibitors (GDIs) sequester Rho GTPases in the inactive state by inhibiting the dissociation of inactive guanine nucleotides [9]. Our previous work showed that Rho, Rac1 and Cdc42 are important effectors of GC malignant transformation and metastasis [10–14]. However, the regulation of Rho GTPases in GC is not yet fully understood.

Rho GEFs include Dbl-related classical GEFs and Dock family atypical Rho GEFs. Since the first GEF-Dbl/MCF2 was reported in 1984, approximately 81 GEFs have been identified, and they have been reported to be involved in various cancers and pathologies [15, 16]. Dock family proteins contain 11 GEFs and are divided into four subfamilies [17, 18]. The Dock-A subfamily consists of Dock1/Dock180, Dock2 and Dock5 [19, 20]. The Dock-B subfamily contains Dock3 and Dock4 [15, 21]. The Dock-C subfamily includes Dock6, Dock7 and Dock8 [16–18, 22]. The Dock-D subfamily is composed of Dock9, Dock10 and Dock11 [15, 23, 24]. As members of the Dock-C family, Dock7 was reported to promote the metastasis of tumors including GBM tumor cells, Esophageal Squamous Cell Carcinoma (ESCC) and glioblastoma [25–27]; Dock6 has been reported to be involved in polarized axon growth; and Dock8 can increase the motility of immune cells [28, 29]; however, the expression profile and the role of Dock6 and Dock8 in tumors remains unknown.

MicroRNAs are a class of small non-coding RNAs that bind to the 3’-UTR of target genes and post-transcriptionally regulate target mRNAs, resulting in changes in the expression of the target genes [30]. MiR-148b can inhibit the metastasis of nasopharyngeal carcinoma [31], lung cancer [32], hepatocellular carcinoma [33], and pancreatic cancer [34]. miR-148b has been reported to be down-regulated in GC and to inhibit its proliferation [35–37]; however, whether it plays a role in GC metastasis remains unclear.

In this study, we showed that Dock6 was highly expressed in GC tissues and that its over-expression was positively correlated with lymph node metastasis and poor prognosis. As a Rho GEF, Dock6 promoted GC migration and invasion by increasing the activation of Rac1 and Cdc42. miR-148b-3p expression was low in GC tissues, and it inhibited GC metastasis by inhibiting the Dock6/Rac1/Cdc42 signaling pathway. The Dock6-Rac1/Cdc42 axis might be a promising target for GC treatment.

## Methods

### Cell culture

The human normal gastric epithelial cell line GES-1 and the GC cell lines AGS, HGC-27, MGC-803, SGC-7901 and BGC-823 were purchased from Genechem (Shanghai, China). GC cell lines with high metastatic potential (MKN-28 M and SGC-7901 M) and corresponding cell lines with low metastatic potential (MKN-28NM and SGC-7901NM) were constructed from the human GC cell lines MKN28 and SGC-7901, respectively, as described previously [3, 4]. All cells were cultured in RPMI-1640 medium (GIBCO, Carlsbad, CA, USA) supplemented with 10% FBS, 100 U/ml penicillin sodium and 100 μg/ml streptomycin at 37 °C in a 5% CO2 incubator.

### Tissue collection

This study was approved by the Medical Ethics Committee of Xijing Hospital (Xi’an, China). Thirty pairs of primary GC tissues and adjacent nontumor tissues were obtained from patients who underwent surgery at Xijing Hospital between 2015 and 2016. All tissues used were verified clinically and pathologically by the Department of Pathology at Xijing Hospital. Informed consent was obtained from all patients involved. Upon removal from the patients, the tissues were frozen in liquid nitrogen and used for total RNA extraction.

### RNA extraction and real-time PCR

Total RNA was extracted with a Takara MiniBEST Universal RNA Extraction Kit (Takara, Dalian, China) according to the manufacturer’s instructions. Reverse transcription and real-time PCR analysis were performed with a Takara PrimeScript RT Reagent Kit and Takara SYBR Green PCR Kit. PCR primers for Dock6, ACTIN, miR-148b-3p and U6 were designed and synthesized by Takara. The primer sequences are listed in Additional file 1: Table S6. The real-time PCR protocol was described previously [38, 39]. GAPDH and the small nuclear RNA U6 were used as internal controls for the mRNA and microRNA analyses, respectively. All reactions were performed in triplicate.

### IHC and ISH of GC tissue microarrays

All tissue microarrays used in this study were purchased from Shanghai Outdo Biotech Co., Ltd. (Shanghai, China). The HStm-Ade180Sur-03 GC tissue microarrays contained 90 pairs of primary GC tissues and paired nontumor tissues and were used for Dock6 (Sigma, HPA049423) and miR-148b-3p staining. The HStm-Ade120lym-01 GC tissue microarray contained 32 pairs of nontumor tissues, paired primary GC tissues and lymph node metastases, and this microarray was used for Dock6 staining. The miR-148b-3p probe was purchased from Exiqon, and the probe (50 nM) was detected with a digoxigenin antibody (Roche, 11,093,274, 1:1000). The IHC and ISH assays were performed as previously described [38]. The IHC and ISH results were analyzed independently by two pathologists who were blinded to the study. The results were scored based on the intensity and the extent of staining. Staining intensity was scored as 0 (negative staining), 1 (weak staining), 2 (moderate staining) and 3 (strong staining). The staining extent was scored based on the percentage of positive cells and was graded as 0 (negative), 1 (0.01–25%), 2 (25.01–50%), 3 (50.01–75%), and 4 (75.01–100%). The histologic score (H score) for each section was calculated with the following formula: histologic score = proportion score×intensity score. Thus, the total score could be 0, 1, 2, 3, 4, 6, 8, 9, or 12, and the staining could be classified as negative (0, 1, 2, 3, 4) or positive (6, 8, 9, 12).

### The clinical information of GC patients in the Kaplan-Meier plotter database

The correlation between Dock6, Dock7 or Dock8 expression and the overall or recurrence-free survival of GC patients was analyzed with data extracted from the Kaplan-Meier plotter database [40]. Gene expression data and relapse-free and overall survival information from the Kaplan-Meier plotter database were downloaded from GEO (Affymetrix microarrays only), EGA and TCGA. The Kaplan-Meier plotter gastric cancer database includes the clinical information of the GSE29272 (*n* = 268), GSE14210 (*n* = 146), GSE22377 (*n* = 43), GSE15459 (*n* = 200), GSE51105 (*n* = 94) and GSE62254 (*n* = 300) data sets. The database is handled by a PostgreSQL server, which integrates gene expression and clinical data simultaneously. To analyze the prognostic value of a particular gene, the patient samples were split into two groups according to the median survival of the patients.

### Plasmid construction and transfection

Lentiviruses for Dock6 over-expression and down-regulation, lentivirus for miR-148b-3p over-expression and control lentiviruses were purchased from Genechem (Shanghai, China). SiRNA for Dock6 and the microRNA mimic and inhibitor for miR-148b-3p were purchased from Ribo Bio (Guangzhou, China). To generate stable Dock6-knock-down cells, SGC-7901 M cells were infected with lenti-Cas9 virus (MOI = 50) and cultured with 2.5 μg/ml puromycin for 2 weeks. Then, the SGC-7901-LV-Cas9 cells were infected with lenti-Dock6-sgRNAs (MOI = 50) or lenti-sgcontrol (MOI = 50). The infection efficiency was confirmed, and the SGC-7901-LV-Cas9-sgDock6 (SGC-7901 M-Dock6-KD) cells or SGC-7901-LV-Cas9-sgcontrol (SGC-7901 M-control) cells were selected by flow cytometry (FCM). To generate Dock6-over-expressing and miR-148b-3p-over-expressing cells, cells were infected with lenti-Dock6 (MOI = 100) or lenti-miR-148b-3p (MOI = 50) or the corresponding lenti-control lentivirus, and stable cells were selected with 2.5 μg/ml puromycin treatment for 2 weeks. The siRNA for Dock6 was used at a final concentration of 100 nM. The microRNA mimic and inhibitor were added to the cells at a final concentration of 150 nM and 300 nM, respectively. Forty-eight hours after transfection with siRNA or microRNA mimic or inhibitor, total RNA and protein were extracted from cells for further study.

### GST pull-down assay and western blot assay

GST pull-down assays were performed with an Active Rac1 Pull-Down and Detection Kit (Thermo Scientific, USA, 16118) according to the manufacturer’s instructions. Western blot assays were performed as described previously [39]. The primary antibodies used were anti-Dock6 (Sigma, HPA049423, 1:1000), anti-β-actin (Proteintech, 60,008–1, 1:2000), anti-Cdc42 (CST, 2462S, 1:1000) and anti-Rac1 (Millipore, 05–389, clone 23A8, 1:200).

### Transwell studies

For migration assays, 5 × 10^4^ cells in 200 μl of RPMI-1640 medium (containing 1% FBS) were seeded into the upper Boyden chambers (Corning, NY, USA). For invasion assay, the chambers were coated with 60 μl of Matrigel (200 mg/ml) and dried overnight under sterile conditions, and 1 × 10^5^ cells in 200 μl of RPMI-1640 medium (containing 1% FBS) were seeded into the chambers. The lower chambers were filled with RPMI-1640 medium (containing 20% FBS), and the cells were incubated for 24 h. Then, the cells remaining on the upper side of the membrane were removed, and the cells adhering to the lower side of the membrane were fixed and stained with 0.5% crystal violet. To evaluate the motility of the cells, cells in 5 random fields (20×) were counted and analyzed. Each assay was performed in triplicate.

### In vivo studies

BALB/C nude mice (6 weeks old) were used for in vivo studies. The mice were housed in the Experimental Animal Center of the Fourth Military Medical University according to the institutional guidelines for animal care. All animal experiments were approved by the Fourth Military Medical University Animal Care Committee. In the tail vein metastatic model, 3 × 10^6^ cells were injected into the tail veins of the nude mice. In the liver metastatic model, 3 × 10^6^ cells were injected into the spleens of the nude mice. Ten weeks after the injection, the mice were injected intraperitoneally with D-luciferin (Xenogen, Hopkinton, MA, 150 mg/kg), and bioluminescence images were taken with an IVIS 100 Imaging System (Xenogen). Then, the mice were sacrificed by cervical dislocation, and the lungs or livers were removed and prepared for histological examination (hematoxylin and eosin (H&E) staining).

### Luciferase reporter study

Plasmids containing the wild-type (WT) Dock6–3’-UTR sequence and a mutant Dock6–3’-UTR sequence were synthesized by GeneCopoeia (Shanghai, China). Luciferase activity was detected with a Dual Luciferase Assay kit (GeneCopoeia) according to the manufacturer’s instructions as described previously [38].

### Statistical analysis

SPSS 20.0 software (SPSS Inc., Chicago, IL, USA) was used for all statistical analyses. The quantitative data were compared between groups with Student’s t-test. The chi-squared test was used to determine whether there was a significant difference in the distribution of Dock6/miR-148b-positive samples between different categories. The overall survival rates were estimated using Kaplan-Meier analyses and the log-rank test. The Cox proportional hazards model was used to determine the independent factors that influence survival based on the variables that had been selected from the univariate analyses. A *P* value of *P* < 0.05 was considered to be statistically significant.

## Results

### Dock6 expression is increased in GC tissues and indicates poor prognosis

To explore the potential roles of the Dock-C subgroup GEFs (Dock6, Dock7 and Dock8) in GC, we first investigated their mRNA expression in 8 GC cohorts (*n* > 10) from the Oncomine database (www.oncomine.org), and the results indicated that Dock6 was the most up-regulated gene (Additional file 2: Table S1). The correlation between Dock6, Dock7 and Dock8 expression and the prognosis of GC patients was then analyzed by Kaplan-Meier analyses [40]. All three probes for Dock6 indicated that patients with high Dock6 expression had shorter overall or recurrence-free survival periods than those with low Dock6 expression, while different probes for Dock7 or Dock8 showed different correlation between high Dock7/Dock8 expression and the overall or recurrence-free survival periods (Additional file 3: Figure S1). Thus, among the Dock-C subgroup Rho GEFs, Dock6 might be an important effector of GC initiation and progression.

**Fig. 1:**
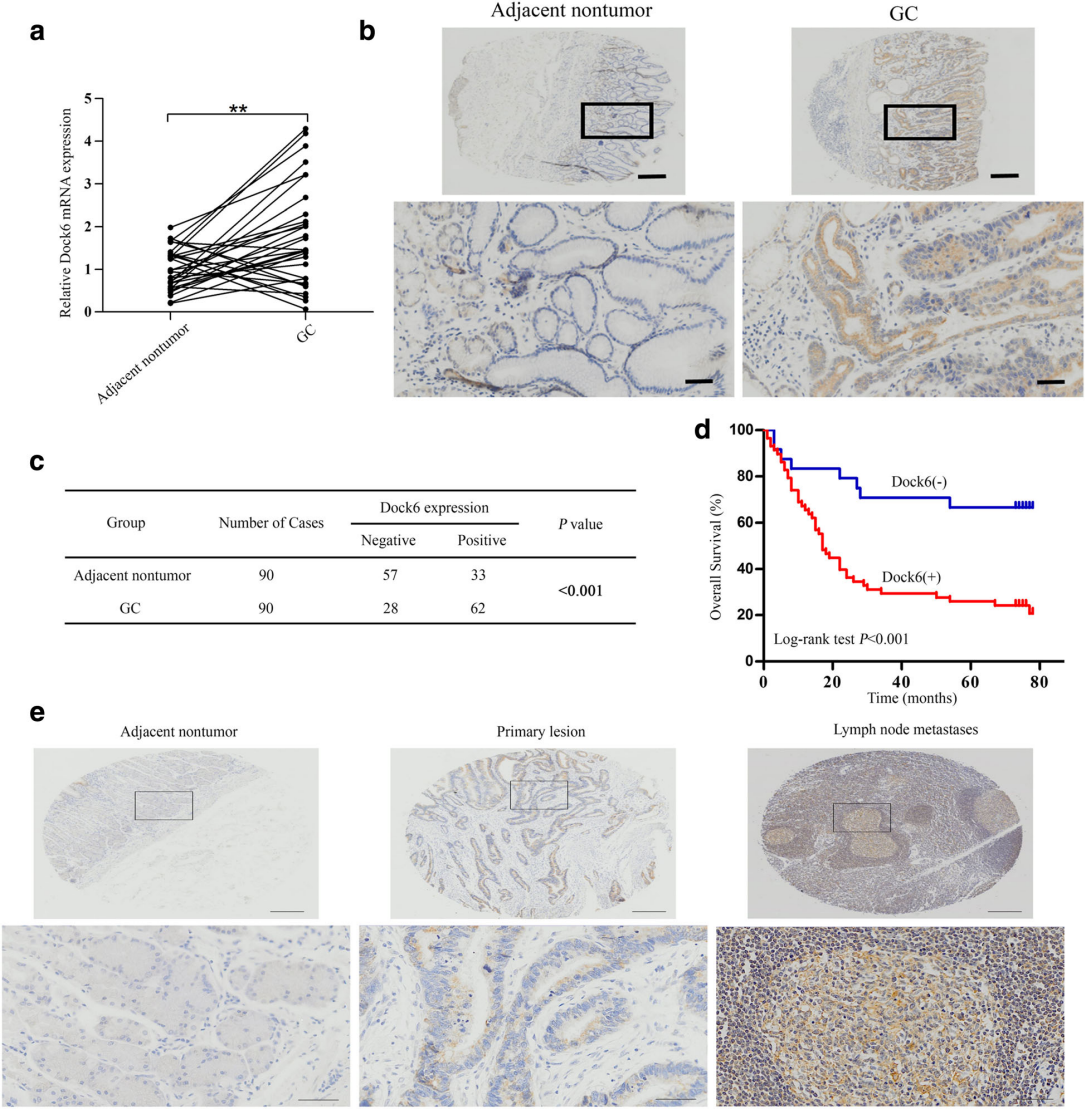
Dock6 is highly expressed in GC tissues and indicates poor prognosis. **a** Relative mRNA expression of Dock6 in primary GC tissues and paired nontumor tissues (*n* = 30), ^**^P < 0.01. **b** Representative expression of Dock6 in GC and adjacent nontumor tissues as detected by IHC. Scale bars represent 200 μm (low magnification) and 50 μm (high magnification). **c** Comparison of Dock6 expression in primary GC tissues and adjacent nontumor tissues. **d** Kaplan-Meier analysis of the correlation between Dock6 expression and the overall survival of GC patients. **e** Representative IHC staining of Dock6 expression in adjacent nontumor tissues, primary GC tissues, and lymph node metastases. Scale bars represent 200 μm (low magnification) and 50 μm (high magnification)

To investigate the potential role of Dock6 in GC, we analyzed the mRNA expression levels of Dock6 in 30 pairs of GC tissues and adjacent nontumor tissues, and real-time PCR results showed that Dock6 mRNA expression was significantly higher in GC tissues than in nontumor tissues (Fig. 1a, *P*< 0.01). IHC staining was then used to examine the Dock6 protein expression levels in 90 pairs of GC tissues and adjacent nontumor tissues, and a higher level of Dock6 was observed in 68.9% (62 in 90) of the GC tissues, while Dock6 was positively expressed in 36.7% (33 in 90) of the nontumor tissues (Fig. 1b and c, *P*< 0.001). Positive Dock6 expression was associated with gender, lymph node metastasis and a higher TNM stage (Additional file 4: Table S2). Univariate and multivariate analyses indicated that a higher clinical stage, positive lymph node metastasis and positive Dock6 expression were independent risk factors for the overall survival of GC patients (Additional file 5: Table S3).Kaplan-Meier analysis results showed that GC patients with positive Dock6 expression exhibited a shorter overall survival time than patients with negative Dock6 expression (Fig. 1d). Furthermore, Dock6 expression was much higher in lymph node metastases than in the primary GC tissues as determined by IHC studies (Fig. 1e). These results suggested that Dock6 may contribute to GC metastasis and malignant progression.

### Dock6 promotes GC proliferation and metastasis

**Fig. 2:**
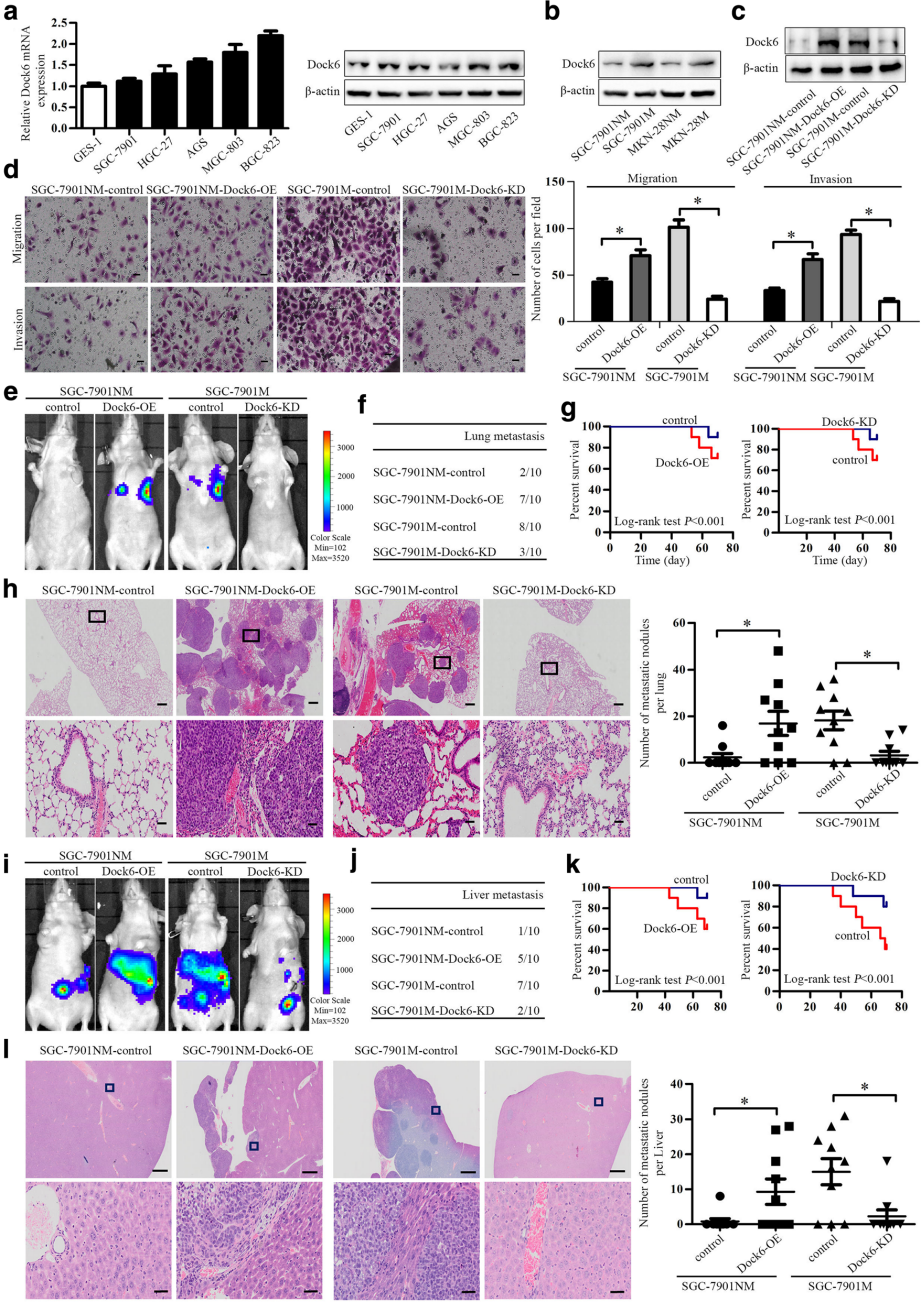
Dock6 promotes GC metastasis in vitro and in vivo. **a** Real-time PCR analysis of Dock6 mRNA expression and western blot analysis of Dock6 protein expression in GES cells and GC cell lines. **b** Western blot analysis of Dock6 protein expression in GC cell lines with high metastatic potential (MKN-28 M and SGC-7901 M) and corresponding cell lines with low metastatic potential (MKN-28NM and SGC-7901NM). **c** Western blot analysis of Dock6 protein expression in the indicated GC cell lines. **d** Dock6 deficiency decreased the migration and invasion abilities of SGC-7901 M cells, while Dock6 over-expression increased the motility of SGC-7901NM cells as determined by transwell studies. Scale bars, 50 μm. ^*^P < 0.05. **e**-**h** In vivo tail vein metastatic assay. The indicated GC cells were injected into the tail vein of nude mice, followed by noninvasive bioluminescence imaging and H&E staining of the lung tissues at 10 weeks after the injection. Scale bars represent 500 μm (low magnification) and 50 μm (high magnification). ^*^P < 0.05. **i-l** In vivo spleen metastatic assay. The indicated GC cells were injected into the spleen of nude mice, followed by noninvasive bioluminescence imaging and H&E staining of liver tissues at 10 weeks after the intrasplenic transplantation. Scale bars represent 500 μm (low magnification) and 50 μm (high magnification). ^*^P < 0.05

Real-time PCR studies and western blot studies were used to investigate the expression of Dock6 in normal gastric epithelial cells (GES-1) and GC cell lines (AGS, SGC-7901, HGC-27, MGC-803 and BGC-823), and Dock6 expression in GC cells was higher than that in GES-1 cells (Fig. 2a). To investigate the role of Dock6 in the proliferation of GC cells, we generated SGC-7901-Dock6-OE cells with the lentivirus Lenti-Dock6 and BGC-823-siDock6 cells with siRNA (Additional file 6: Figures S2a-b). MTT assays and colony formation studies showed that Dock6 over-expression increased the proliferation of SGC-7901 cells, while Dock6 down-regulation decreased the proliferation of BGC-823 cells (Additional file 6: Figures S2c-d). FCM analyses showed that Dock6 over-expression increased the proportion of cells in G2/M stage, while Dock6 RNAi decreased the proportion of cells in G2/M stage (Additional file 6: Figure S2e). These results suggested that Dock6 contributed to the proliferation of GC cells.

To determine the possible roles of Dock6 in the migration and invasion of GC cells, Dock6 expression in GC cell lines with high metastatic potential (MKN-28 M and SGC-7901 M) and corresponding cell lines with low metastatic potential (MKN-28NM and SGC-7901NM) was detected by western blot studies. Dock6 expression was much higher in MKN-28 M and SGC-7901 M cells than in MKN-28NM and SGC-7901NM cells, respectively (Fig. 2b). SGC-7901NM-Dock6-OE cells were then established by Lenti-Dock6 infection, and SGC-7901 M-Dock6-KD cells were established by infection with a lentivirus expressing Cas9 and Dock6-sgRNA, and the Dock6 protein expression levels in these cell lines were then detected by western blot (Fig. 2c). The results of transwell studies showed that Dock6 over-expression up-regulated the migration and invasion of SGC-7901NM cells, while Dock6 down-regulation decreased the motility of SGC-7901 M cells (Fig. 2d).

To investigate the roles of Dock6 in the metastasis of GC cells in vivo, in vivo tail vein and liver metastatic assays were used. In the tail vein metastatic assay, SGC-7901NM-Dock6-OE cells, SGC-7901 M-Dock6-KD cells or control cells were injected into the tail veins of nude mice (10 mice per group). Ten weeks after the injection, bioluminescence images were taken (Fig. 2e). Dock6 over-expression increased the lung metastasis of SGC-7901NM cells and reduced the overall survival time of the mice, while Dock6 down-regulation decreased the lung metastasis of SGC-7901 M cells and increased the overall survival time of the mice (Fig. 2f-g). H&E assays confirmed the incidence of lung metastasis (Fig. 2h). In the liver metastatic assay, SGC-7901NM-Dock6-OE cells, SGC-7901 M-Dock6-KD cells or control cells were injected into the spleens of nude mice (10 mice per group). Ten weeks after the intra-splenic injection, bioluminescence images were taken (Fig. 2i). Dock6 over-expression increased the liver metastasis of SGC-7901NM cells and reduced the overall survival time of the mice, while Dock6 down-regulation decreased the liver metastasis of SGC-7901 M cells and increased the overall survival time of the mice (Fig. 2j-k). H&E studies confirmed the incidence of liver metastasis (Fig. 2l). Taken together, these results show that Dock6 increased the metastasis of GC cells in vitro and in vivo.

### Dock6 promotes GC migration and invasion by activating Rac1 and Cdc42

**Fig. 3:**
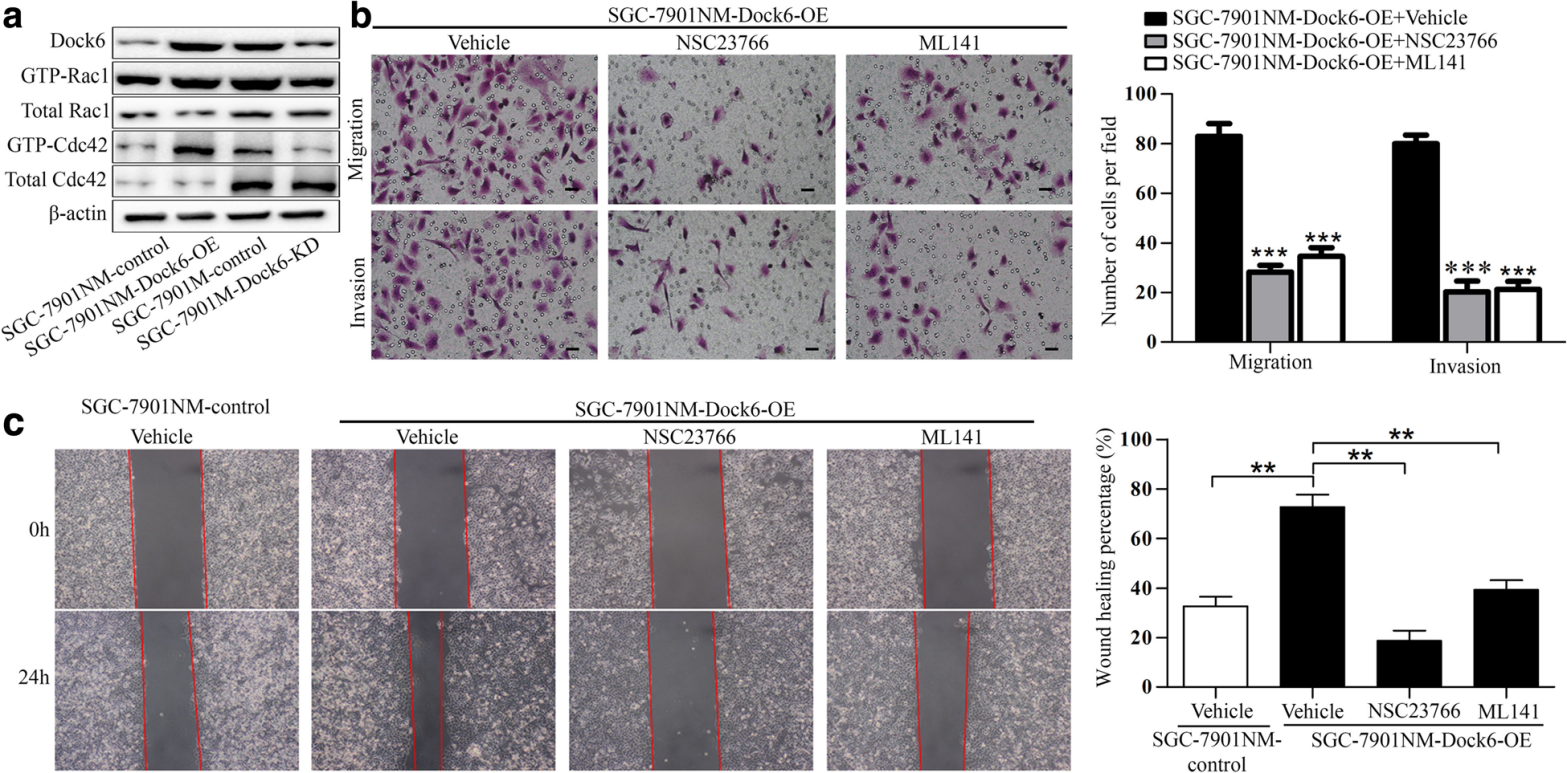
Dock6 promotes GC migration and invasion by activating Rac1 and Cdc42. **a** GST pull-down and western blot analyses of the expression of Dock6 and the activation state and total expression of Rac1/Cdc42 in the indicated cells. **b** A Rac1 inhibitor (NSC23766, 50 μM) and Cdc42 inhibitor (ML141, 20 μM) could block Dock6-mediated cell migration and invasion as detected by transwell studies. Scale bars, 50 μm. **c** Wound healing assays were used to evaluate the migration of the indicated cells

GEFs in the Dock family have two domains: the DHR1 domain binds phospholipids, and the DHR2 domain executes the guanine nucleotide exchange activity [16, 41]. Dock6 has been reported to activate Rac1 and Cdc42 via its DHR2 domain and promote axonal outgrowth and vascular smooth muscle cell (VSMC) migration [41, 42]. We used GST pull-down assays to study the effect of Dock6 on Rac1 and Cdc42 activation in GC cells. Dock6 over-expression increased Rac1 and Cdc42 activation, and Dock6 knock-down decreased the GTP-Rac1 and GTP-Cdc42 levels, while Dock6 did not affect the expression of total Rac1 and Cdc42 (Fig. 3a). Transwell assays showed that the Rac1 inhibitor NSC23766 (Selleck, 50 μM) and Cdc42 inhibitor ML141 (Selleck, 20 μM) blocked Dock6-mediated GC migration and invasion (Fig. 3b). Wound healing experiments showed that Dock6 over-expression up-regulated the migration ability of SGC-7901NM cells, while NSC23766 and ML141 could reverse Dock6-mediated cell migration (Fig. 3c), suggesting that Dock6 contributed to GC metastasis by increasing the activation of Rac1 and Cdc42.

### Hsa-miR-148b-3p inhibits the expression of Dock6 by targeting its 3’-UTR

**Fig. 4:**
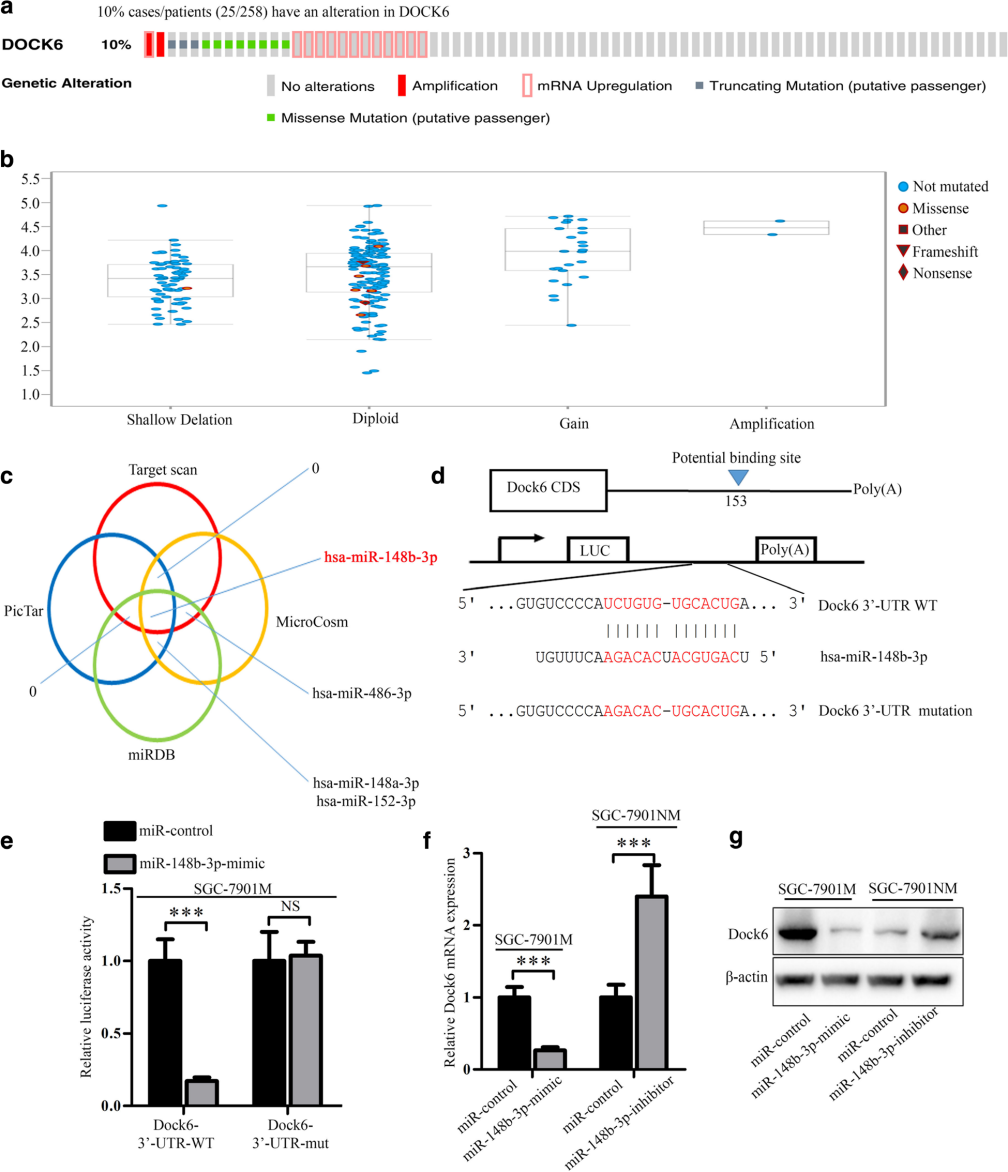
Hsa-miR-148b-3p inhibits the expression of Dock6 by binding to its 3’-UTR. **a** Genetic alteration rates of Dock6 in GC patients in a TCGA cohort. **b** Dock6 mRNA expression patterns in GC patients with different genetic alterations in a TCGA cohort. **c** The Target scan (red), Microcosm (yellow), miRDB (green) and PicTar (blue) databases were used to predict the microRNAs that could target Dock6. **d** Schematic of the predicted miR-148b-3p binding sites in the 3’-UTR of Dock6 and the mutant binding sites. **e** miR-148b-3p inhibits Dock6 transcription. Cells were co-transfected with the Dock6–3’-UTR, a mutated Dock6–3’-UTR or a positive control and a miR-control or miR-148b-3p mimic, and the relative luciferase activity was determined. ^*^P < 0.05, ^***^P < 0.001. **f** Real-time PCR analysis of Dock6 mRNA expression in the indicated cells. ^***^P < 0.001. **g** Western blot analysis of Dock6 protein expression in the indicated cells

By analyzing the gene mutation and copy number of Dock6 in the TCGA database [43, 44], we found that Dock6 was amplified in 2/258 (0.8%) patients (Fig. 4a), and Dock6 mRNA expression was positively correlated with changes in copy number (Fig. 4b). However, among patients with high Dock6 mRNA expression, 12 out of 258 (4.7%) did not exhibit gene amplification, indicating that alternative mechanisms are involved in its up-regulation.

MicroRNAs are important effectors of cancer progression. We next investigated whether microRNAs play a role in the up-regulation of Dock6 expression in GC. The Target scan, Microcosm, miRDB and PicTar databases were used to predict the microRNAs that could target Dock6. Only Hsa-miR-148b-3p was identified by all four databases, indicating that miR-148b-3p could regulate Dock6 expression (Fig. 4c, Additional file 7: Table S4). To further investigate whether miR-148b-3p regulated the expression of Dock6 by binding to its 3’-UTR, a luciferase reporter assay was performed. The WT Dock6 3’-UTR sequence or a mutant 3’-UTR sequence was inserted into a luciferase reporter vector (Fig. 4d). Each construct was co-transfected with a miR-mimic control or a miR-148b-3p mimic, and the results showed that the miR-148b-3p mimic significantly decreased the luciferase activity in cells transfected with the WT Dock6 3’-UTR, but not in cells transfected with the mutant Dock6 3’-UTR (Fig. 4e), suggesting that miR-148b-3p could decrease Dock6 transcription by directly binding to its 3’-UTR. Real-time PCR and western blot assays were then used to validate the regulation of Dock6 expression by miR-148b-3p, and the results showed that the miR-148b-3p mimic decreased the expression of Dock6, while a miR-148b-3p inhibitor increased the expression of Dock6 (Fig. 4f-g).

### Dock6 expression is negatively correlated with miR-148b-3p expression in human GC tissues

**Fig. 5:**
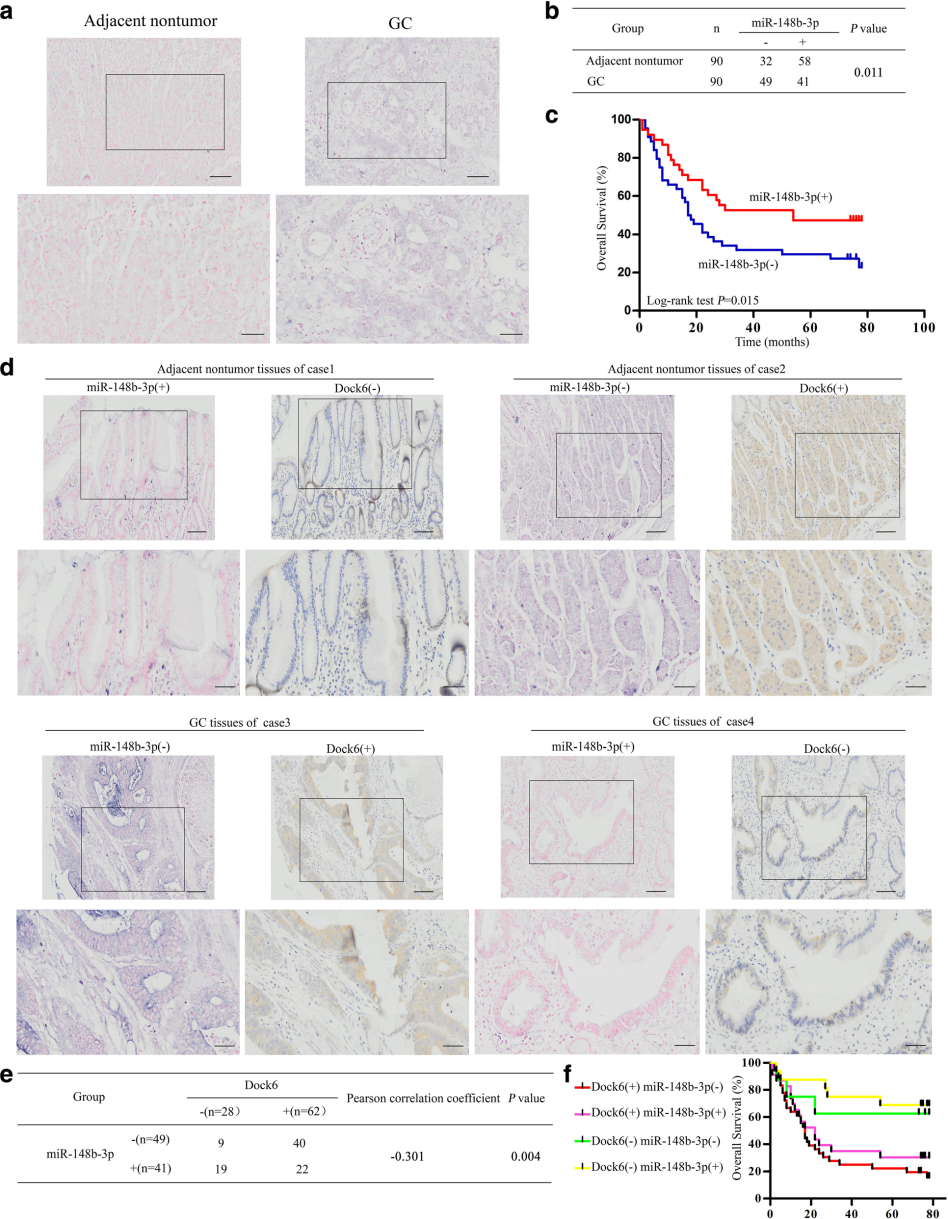
Dock6 expression is negatively correlated with miR-148b-3p expression in human GC tissues. **a** Representative expression of miR-148b-3p in GC tissues and adjacent nontumor tissues as detected by in situ hybridization. Scale bars represent 100 μm (low magnification) and 50 μm (high magnification). **b** Comparison of miR-148b-3p expression in primary GC tissues and adjacent nontumor tissues. **c** Kaplan-Meier analysis of the correlation between miR-148b-3p expression and the overall survival of GC patients. **d** Representative IHC staining of Dock6 and in situ hybridization staining of miR-148b-3p in the indicated adjacent nontumor tissues and primary GC tissues. Scale bars represent 100 μm (low magnification) and 50 μm (high magnification). **e** The correlation between Dock6 expression and miR-148b-3p expression in the same GC tissues were analyzed. **f** Kaplan-Meier analysis of the correlation between concurrent Dock6 and miR-148b-3p expression and the overall survival of GC patients

miR-148b-3p has been reported to be expressed at a low level in GC tissues and to inhibit GC proliferation [36, 37]. To validate the role of miR-148b-3p in GC, we first examined miR-148b-3p expression in GC tissues and paired nontumor tissues by in situ hybridization. miR-148b-3p expression was significantly higher in the nontumor tissues than in the GC tissues (Fig. 5a-b). Negative expression of miR-148b-3p in GC tissues was positively correlated with lymph node metastasis and a higher TNM stage (Additional file 8: Table S5). Kaplan-Meier analysis results showed that GC patients with negative miR-148b-3p expression had a shorter overall survival than patients with positive miR-148b-3p expression (Fig. 5c). Dock6 expression in GC tissues was negatively correlated with the expression of miR-148b-3p (Pearson correlation coefficient = **−** 0.301, *P* = 0.004) (Fig. 5d-e). Kaplan-Meier analyses showed that GC patients with positive Dock6 expression and negative miR-148b-3p expression had the shortest overall survival (Fig. 5f). Thus, Dock6 expression was negatively correlated with miR-148b-3p expression in human GC tissues.

### miR-148b-3p inhibits GC metastasis by inhibiting the Dock6/Rac1/Cdc42 signaling pathway

**Fig. 6:**
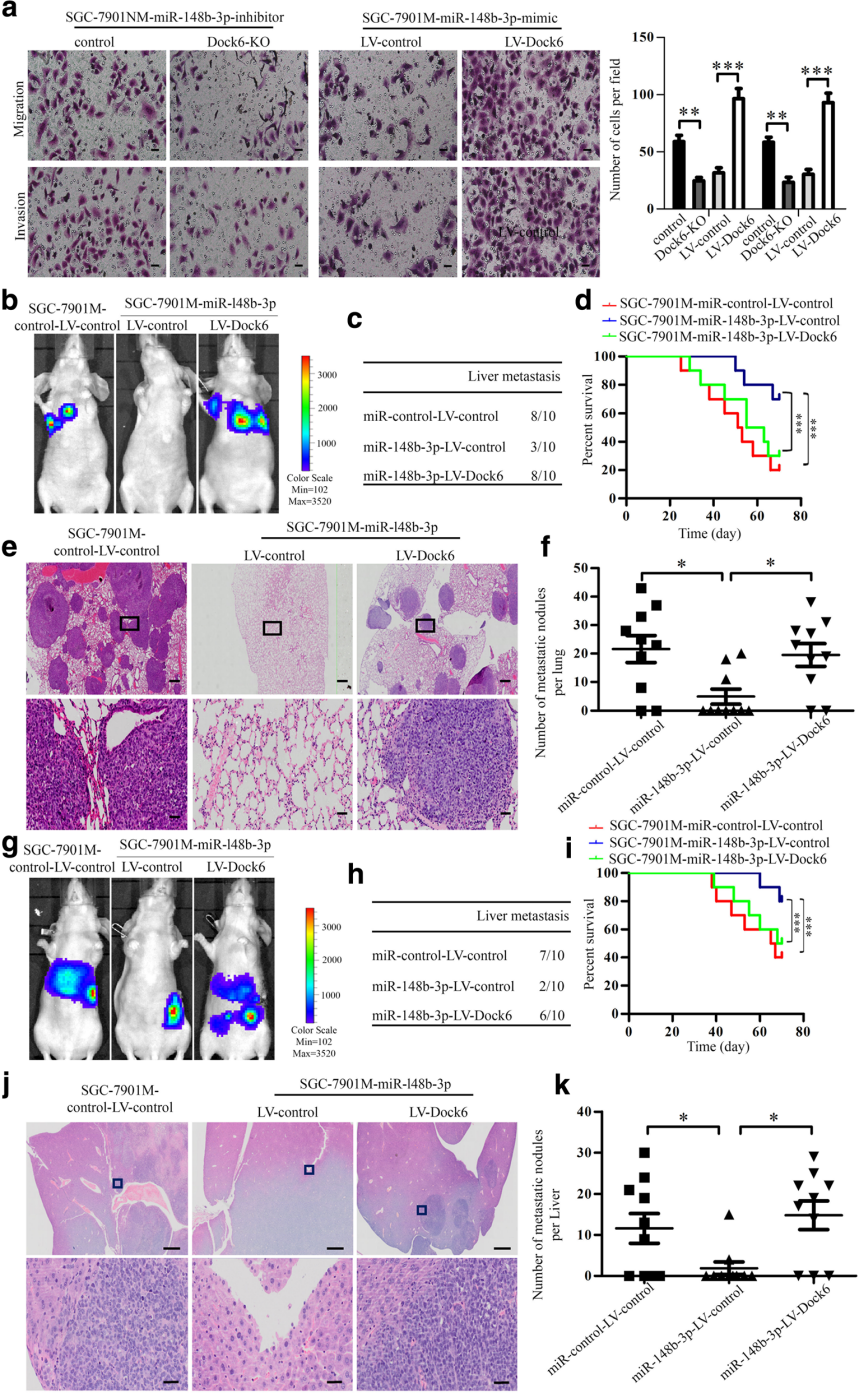
miR-148b-3p inhibits GC metastasis by decreasing the expression of Dock6. **a** Transwell assay analyses of the migration and invasion abilities of the indicated GC cells. Scale bars, 50 μm. ^**^P < 0.01, ^***^P < 0.001. **b-f** In vivo tail vein metastatic assay. The indicated GC cells were injected into the tail vein of nude mice, followed by noninvasive bioluminescence imaging and H&E staining of lung tissues at 10 weeks after the injection. Scale bars represent 500 μm (low magnification) and 50 μm (high magnification). ^*^P < 0.05. **g-k** In vivo spleen metastatic assay. The indicated GC cells were injected into the spleen of nude mice, followed by noninvasive bioluminescence imaging and H&E staining of liver tissues at 10 weeks after the intrasplenic transplantation. Scale bars represent 500 μm (low magnification) and 50 μm (high magnification). ^*^P < 0.05

miR-148b-3p has been reported to inhibit the metastasis of various cancers [32–34]; however, its role in GC metastasis remains unclear. We next investigated whether miR-148b-3p regulated GC metastasis by inhibiting Dock6 expression. Transwell assays showed that Dock6 over-expression reversed the miR-148b-3p mimic-mediated inhibition of SGC-7901 M cell migration and invasion, while Dock6 down-regulation blocked the miR-148b-3p inhibitor-mediated increase in SGC-7901 M cell migration and invasion (Fig. 6a). In vivo tail vein and liver metastatic studies showed that miR-148b-3p over-expression decreased the lung and liver metastasis of SGC-7901 M cells, while Dock6 over-expression reversed this metastatic inhibition (Fig. 6b-j).

**Fig. 7:**
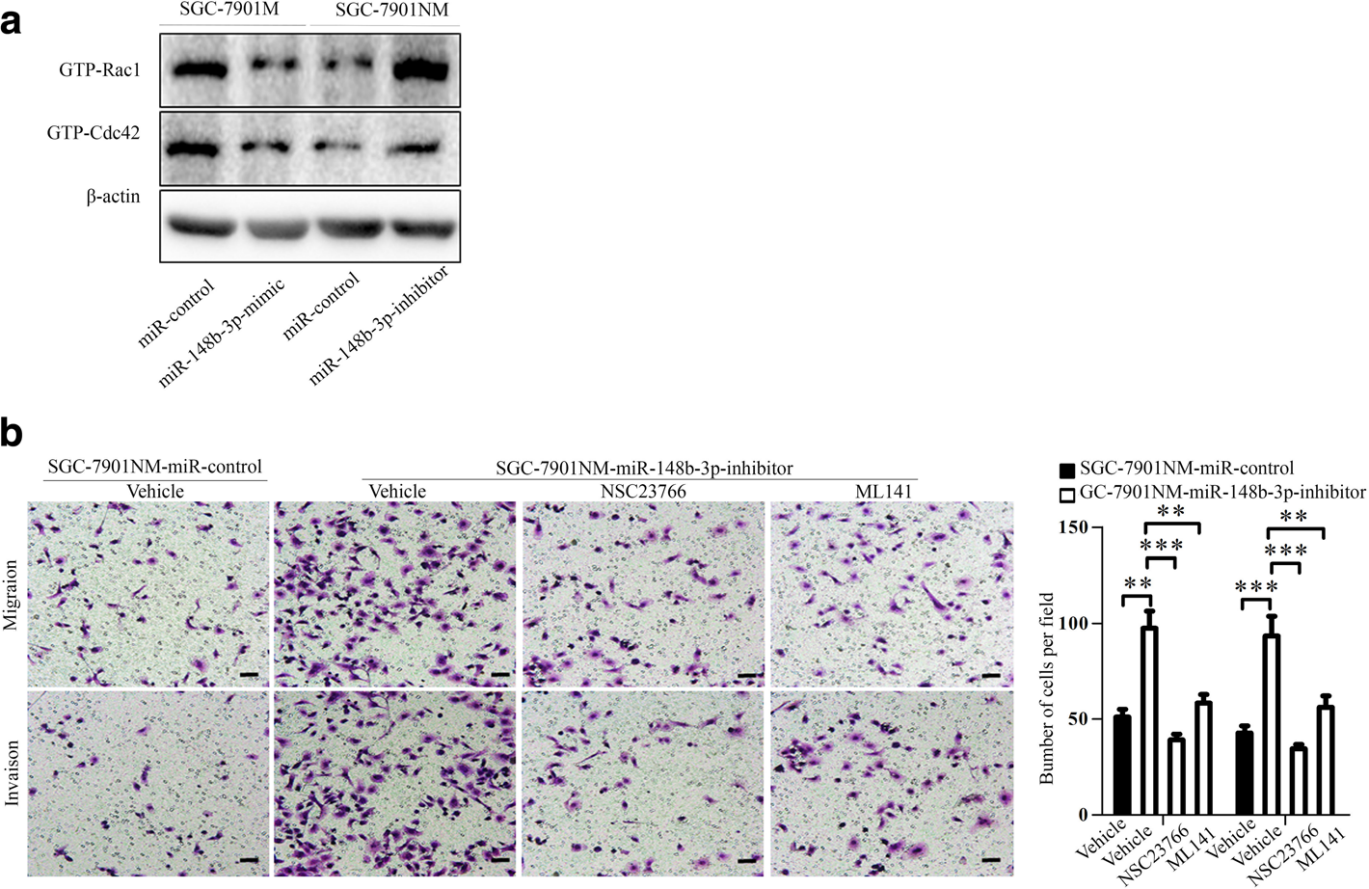
miR-148b-3p promotes the motility of GC cells by activating Rac1 and Cdc42. **a** GST pull-down and western blot analysis of the expression levels of GTP-Rac1 and GTP-Cdc42 in the indicated GC cells. **b** A miR-148b-3p inhibitor increased the migration and invasion abilities of SGC-7901NM cells, while a Rac1 inhibitor (NSC23766) and Cdc42 inhibitor (ML141) blocked the miR-148b-3p inhibitor-mediated GC cell migration and invasion as detected by transwell studies. Scale bars, 50 μm. **P < 0.01, ***P < 0.001

Finally, GST pull-down results showed that the miR-148b-3p mimic decreased Rac1 and Cdc42 activation, while the miR-148b-3p inhibitor increased the levels of GTP-Rac1 and GTP-Cdc42 (Fig. 7a), suggesting that miR-148b-3p could inhibit the activation of Rac1 and Cdc42. A transwell assay showed that the miR-148b-3p inhibitor up-regulated the migration and invasion of SGC-7901NM cells, while the Rac1 inhibitor NSC23766 and Cdc42 inhibitor ML141 could block miR-148b-3p inhibitor-mediated GC migration and invasion (Fig. 7b). These results indicated that miR-148b-3p decreased the motility of GC cells by inhibiting the Dock6/Rac1/Cdc42 signaling pathway.

## Discussion

Rho GTPases play critical roles in the initiation and progression of various tumors, and our previous data showed that Rho, Rac1 and Cdc42 are important effectors of GC malignant transformation and metastasis [10–14]. However, the regulation of Rho GTPases in GC remains largely unknown. Through bioinformatics analyses, we found that Dock6, an atypical Rho GEF, may be involved in GC progression. Dock6 has been reported to promote axonal outgrowth and VSMC migration [28, 41, 42]. However, the role and molecular mechanism of Dock6 in GC are unknown. In this study, we provide the first evidence that Dock6 is over-expressed in GC and that its positive expression is associated with lymph node metastasis and a higher TNM stage. GC patients with positive Dock6 expression exhibited a shorter overall survival time than patients with negative Dock6 expression. In this study, we found that Dock6 could promote the proliferation of GC cells in vitro. We also found that Dock6 expression in lymph node metastases was higher than that in the primary GC tissues and that Dock6 could increase the migration and invasion abilities of GC cells in vitro and in vivo.

As a member of the Dock family of GEFs, Dock6 has been reported to promote axonal outgrowth and VSMC migration by “turning on” Rac1 and Cdc42 [41, 42]. In accordance with previous reports, our data showed that Dock6 could promote the motility of GC cells by activating Rac1 and Cdc42. Recently, a compensatory mechanism for Dock6 knock-down-mediated Rac1/Cdc42 activation inhibition was reported [45, 46]. Upon the acute knock-down of Dock6 with RNAi, the activation of Rac1 and Cdc42 was decreased, and the activation of RhoA was increased; however, when Dock6 was chronically down-regulated, ISG15 expression was inhibited, and the active states of Rac1 and Cdc42 were stabilized by IQGAP1 [47, 48]. In this study, we found that Rac1 and Cdc42 activation was affected by both chronic down-regulation and chronic up-regulation of Dock6, and therefore whether this compensatory mechanism exists in GC requires further investigation.

In neurons, AKT was found to inactivate Dock6 by increasing its phosphorylation at Ser^1194^, whereas PP2A increased the GEF activity of Dock6 by dephosphorylating Dock6 [28]. miR-142-3p could inhibit the motility of VSMCs by directly targeting Dock6 and decreasing its expression [42]. However, the mechanism of Dock6 over-expression in GC remains unclear. In this study, we reported that Dock6 mRNA expression was positively correlated with changes in its copy number. In addition to the change in copy number, other mechanisms were involved in Dock6 mRNA up-regulation. To investigate whether microRNAs were involved in the expression regulation of Dock6, we used four databases to predict microRNAs that could target Dock6 and found that miR-148b-3p may regulate Dock6 expression. Using luciferase reporter assays, we found that miR-148b-3p inhibited Dock6 transcription by targeting its 3’-UTR. Previously, miR-148b was reported to inhibit GC proliferation [36, 37]. In this study, we found that miR-148b-3p expression was low in GC tissues and that its negative expression in GC tissues indicated poor prognosis. Dock6 expression was negatively correlated with miR-148b-3p expression in GC, and patients with positive Dock6 expression but negative miR-148b-3p expression had the poorest prognosis. We also found that miR-148b-3p inhibited GC metastasis by inhibiting the activation of Rac1 and Cdc42. All these results suggested that miR-148b-3p inhibited GC metastasis by inhibiting the Dock6/Rac1/Cdc42 axis.

## Conclusions

We demonstrated that Dock6 was over-expressed in GC and promoted GC metastasis by activating Rac1 and Cdc42. miR-148-3p decreased GC motility by inhibiting the Dock6/Rac1/Cdc42 signaling pathway. Thus, Dock6 might be a potential prognostic biomarker and a novel therapeutic target for GC.

## Additional files

Additional file 1: **Table S6.** The sequences of siRNAs and primers. (DOCX 17 kb)

Additional file 2: **Table S1.** The expression analysis of Dock6, Dock7 and Dock8 in the Oncomine database (*n* > 10). (DOCX 18 kb)

Additional file 3: **Figure S1.** The correlation between Dock6, Dock7, or Dock8 expression and the survival of GC patients. Three probes for Dock6 (a), Dock7 (b) and Dock8 (c), respectively, were used to predict the correlation between Dock6, Dock7, or Dock8 expression and the overall or recurrence-free survival of GC patients. The survival data were extracted from Kaplan-Meier plotter database (www.kmplot.com). (PDF 3758 kb)

Additional file 4: **Table S2.** Correlation between Dock6 expression and pathological characteristics of GC patients. (DOCX 19 kb)

Additional file 5: **Table S3.** Univariate and multivariate analyses of factors associated with survival of 90 GC patients. (DOCX 19 kb)

Additional file 6: **Figure S2.** Dock6 promotes the proliferation of GC cells. (a-b) The construction of SGC-7901 cells with Dock6 over-expression and BGC-823 cells with Dock6 knock-down. Real-time PCR and western blot analyses of Dock6 mRNA or protein expression in the indicated cells. **P < 0.01, ***P < 0.001. (c) MTT assay analyses of the proliferation of the indicated cells. *P < 0.05. (d) Colony formation of the indicated cells and the number of colonies. *P < 0.05. (e) The cell cycle distribution was analyzed by FCM, and the percentages of cells in different stages were determined. (PDF 1392 kb)

Additional file 7: **Table S4.** microRNAs that could target Dock6 as predicted by Target scan, Microcosm, miRDB and PicTar databases. (DOCX 22 kb)

Additional file 8: **Table S5.** Correlation between miR-148b-3p expression and pathological characteristics of GC patients. (DOCX 19 kb)

## Abbreviations

FCM: Flow cytometry
GAPs: GTPase activating proteins
GC: Gastric cancer
GDIs: Guanine nucleotide dissociation inhibitors
GEFs: Guanine nucleotide exchange factors
GES: Gastric epithelial cells
H&E: Hematoxylin and eosin
IHC: Immunohistochemistry
ISH: In situ hybridization
MDR: Multiple drug resistance
VSMC: Vascular smooth muscle cell
