# Supplementary material for: miR-148b-3p inhibits gastric cancer metastasis by inhibiting the Dock6/Rac1/Cdc42 axis
Source: J Exp Clin Cancer Res. 2018 Mar 27;37:71. doi: 10.1186/s13046-018-0729-z (PMC5872400; doi:10.1186/s13046-018-0729-z)
Supplement: Supplementary file 1 — Table S6. The sequences of siRNAs and primers. (DOCX 17 kb) [file 13046_2018_729_MOESM1_ESM.docx]

**Additional file 1: Table S6.** The sequence of siRNAs and primers

|  | sequence |
| --- | --- |
| **siRNAs** |  |
| siDock6-1 | GTGGAACCGTACTTTGATA |
| siDock6-2 | GTCCAAGCTTGACTCACAA |
| siDock6-3 | GCTGTACCTTTGCCTAGCT |
| **sgRNAs for Dock6 knockout** |  |
| Dock6-sgRNA1 | CCTGTTGATCTTGTGCGCGA |
| Dock6-sgRNA2 | CTGCTTCGAGGGGTGTCTTC |
| Dock6-sgRNA3 | CCTTCGCGCACAAGATCAAC |
| **Primers for real-time PCR:** |  |
| Dock6 sense | CGATCACGAAGCTGGCAGAG |
| Dock6 antisense | TGAGCTCGTAGGTATCAAAGTACGG |
| GAPDH sense | CGAACCTCTCTGCTCCTCCTGTTCG |
| GAPDH antisense | CATGGTGTCTGAGCGATGTGG |
| miR-148b-3p sense | TCAGTGCATCACAGAACTTTGT |
| U6 sense | GGAACGATACAGAGAAGATTAGC |
| U6 antisense | TGGAACGCTTCACGAATTTGCG |
| **microRNA mimics** | |
| miR-148b-3p mimic | UCAGUGCAUCACAGAACUUUGU |
| miR-mimic negative control | UUUGUACUACACAAAAGUACUG |
