# Supplementary material for: miR-148b-3p inhibits gastric cancer metastasis by inhibiting the Dock6/Rac1/Cdc42 axis
Source: J Exp Clin Cancer Res. 2018 Mar 27;37:71. doi: 10.1186/s13046-018-0729-z (PMC5872400; doi:10.1186/s13046-018-0729-z)
Supplement: Supplementary file 2 — Table S1. The expression analysis of Dock6, Dock7 and Dock8 in the Oncomine database (n > 10). (DOCX 18 kb) [file 13046_2018_729_MOESM2_ESM.docx]

**Additional file 2: Table S1.** The expression analysis of Dock6, Dock7 and Dock8 in ONCOMINE database (n>10)

| Data base | Dock6 | | Dock7 | | Dock8 | |
| --- | --- | --- | --- | --- | --- | --- |
|  | P | Fold Change | P | Fold Change | P | Fold Change |
| Derrico (Intestinal Type) | 9.42E-04 | 1.305 | 0.026 | 1.220 | 1.000 | -2.609 |
| Chen (Intestinal Type) | 0.375 | 1.023 | 3.82E-4 | 1.224 | 0.371 | 1.027 |
| Chen (Diffuse Type) | 0.022 | 1.168 | 0.286 | 1.049 | 0.053 | 1.217 |
| Chen (Mixed Type) | 0.202 | 1.148 | 0.003 | 1.301 | 0.250 | 1.130 |
| Cui | 1.99E-5 | 1.261 | 0.495 | 1.001 | 0.056 | 1.230 |
| Wang | 6.25E-4 | 1.467 | 0.252 | 1.089 | 0.704 | -1.170 |
| Cho (Intestinal Type) | 0.010 | 1.410 | 0.252 | 1.016 | 0.884 | -1.205 |
| Cho (Diffuse Type) | 0.021 | 1.331 | 0.424 | 1.021 | 0.999 | -1.483 |
