# Supplementary figures and images for: miR-148b-3p inhibits gastric cancer metastasis by inhibiting the Dock6/Rac1/Cdc42 axis
Source: J Exp Clin Cancer Res. 2018 Mar 27;37:71. doi: 10.1186/s13046-018-0729-z (PMC5872400; doi:10.1186/s13046-018-0729-z)

a

Dock6

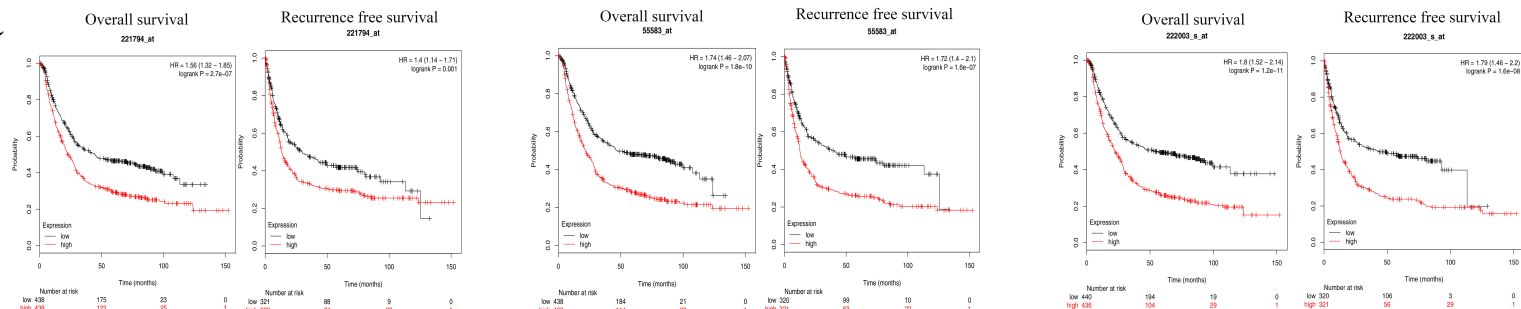

b

Dock7

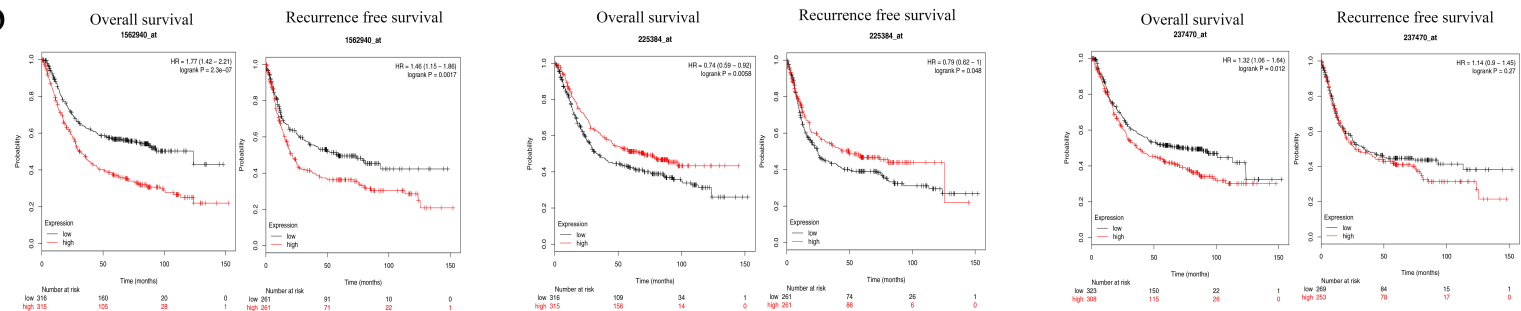

c

Dock8

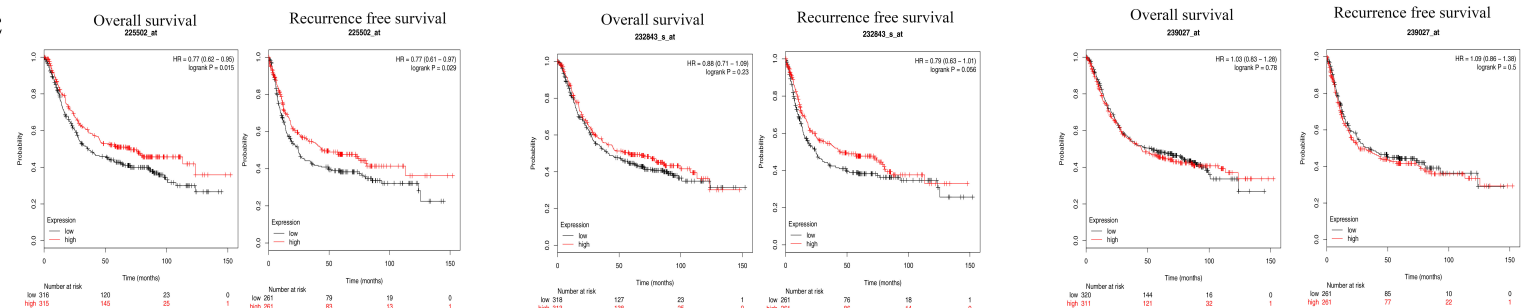

Supplement: Supplementary file 3 — Figure S1. The correlation between Dock6, Dock7, or Dock8 expression and the survival of GC patients. Three probes for Dock6 (a), Dock7 (b) and Dock8 (c), respectively, were used to predict the correlation between Dock6, Dock7, or Dock8 expression and the overall or recurrence-free survival of GC patients. The survival data were extracted from Kaplan-Meier plotter database (www.kmplot.com). (PDF 3758 kb) [file 13046_2018_729_MOESM3_ESM.pdf]

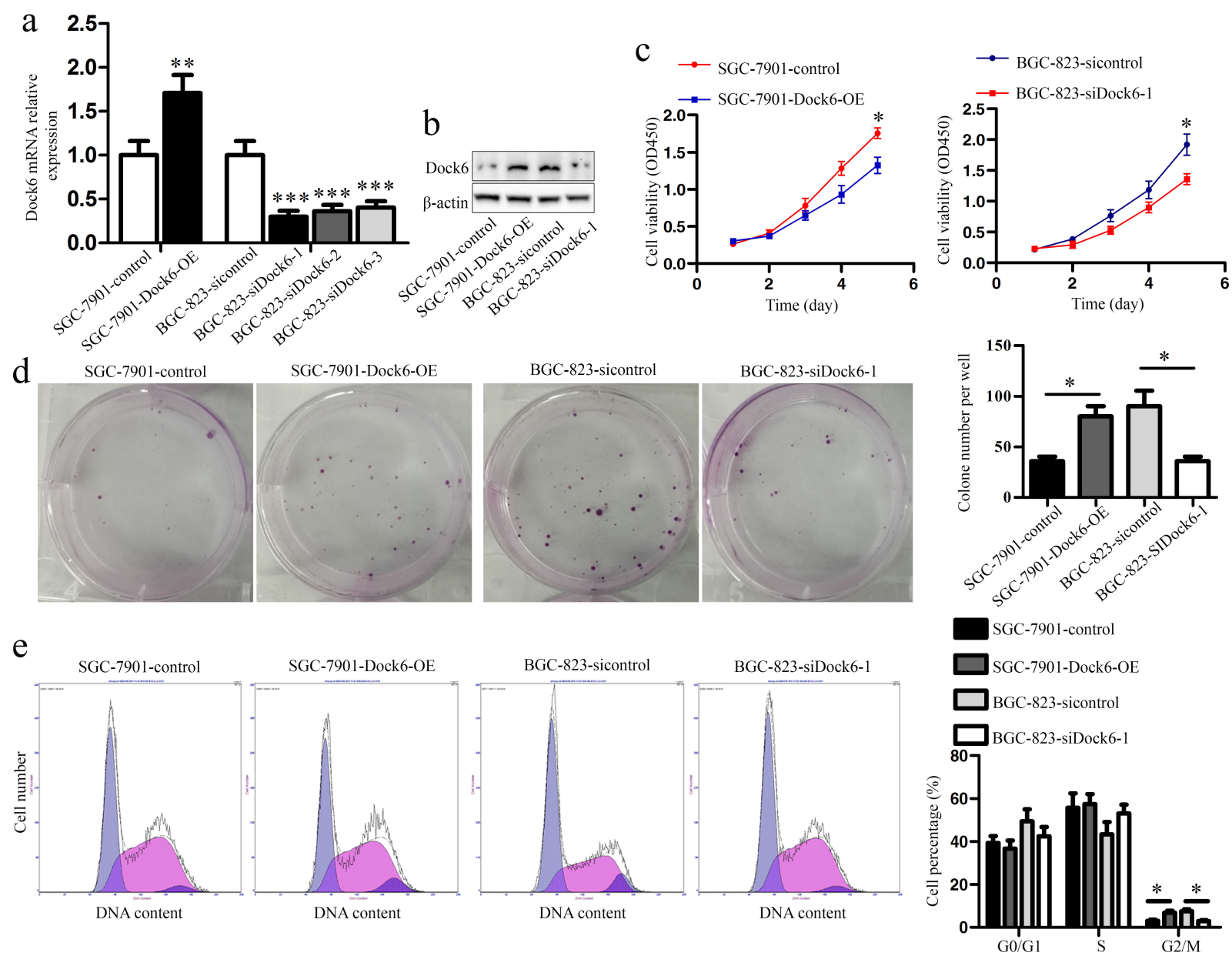

Supplement: Supplementary file 6 — Figure S2. Dock6 promotes the proliferation of GC cells. (a-b) The construction of SGC-7901 cells with Dock6 over-expression and BGC-823 cells with Dock6 knock-down. Real-time PCR and western blot analyses of Dock6 mRNA or protein expression in the indicated cells. **P < 0.01, ***P < 0.001. (c) MTT assay analyses of the proliferation of the indicated cells. *P < 0.05. (d) Colony formation of the indicated cells and the number of colonies. *P < 0.05. (e) The cell cycle distribution was analyzed by FCM, and the percentages of cells in different stages were determined. (PDF 1392 kb) [file 13046_2018_729_MOESM6_ESM.pdf]
