# Supplementary material for: miR-148b-3p inhibits gastric cancer metastasis by inhibiting the Dock6/Rac1/Cdc42 axis
Source: J Exp Clin Cancer Res. 2018 Mar 27;37:71. doi: 10.1186/s13046-018-0729-z (PMC5872400; doi:10.1186/s13046-018-0729-z)
Supplement: Supplementary file 4 — Table S2. Correlation between Dock6 expression and pathological characteristics of GC patients. (DOCX 19 kb) [file 13046_2018_729_MOESM4_ESM.docx]

**Additional file 4: Table S2.** Correlation between Dock6 expression and pathological characteristics of GC patients

| Variables |  | No. | Dock6 (n=90)  -(n=28) +(n=62) | | *P* value |
| --- | --- | --- | --- | --- | --- |
| Age | ≤60 | 32 | 10 | 22 | 0.983 |
|  | >60 | 58 | 18 | 40 |  |
| Gender | Male | 62 | 15 | 47 | **0.035** |
|  | Female | 28 | 13 | 15 |  |
| Max tumor size | ≤5cm | 30 | 13 | 17 | 0.077 |
|  | >5cm | 60 | 15 | 45 |  |
| Differentiation | Well /Moderate | 34 | 13 | 21 | 0.255 |
|  | Poor | 56 | 15 | 41 |  |
| T Classification | T1-T2 | 11 | 8 | 3 | **0.001** |
|  | T3-T4 | 79 | 20 | 59 |  |
| Lymph node metastasis | - | 25 | 14 | 11 | **0.002** |
|  | + | 65 | 14 | 51 |  |
| N classification | N0-N1 | 35 | 19 | 16 | **<0.001** |
|  | N2-N3 | 55 | 9 | 46 |  |
| Clinical stage | I-II | 35 | 19 | 16 | **<0.001** |
|  | III-IV | 55 | 9 | 46 |  |
