# Supplementary material for: miR-148b-3p inhibits gastric cancer metastasis by inhibiting the Dock6/Rac1/Cdc42 axis
Source: J Exp Clin Cancer Res. 2018 Mar 27;37:71. doi: 10.1186/s13046-018-0729-z (PMC5872400; doi:10.1186/s13046-018-0729-z)
Supplement: Supplementary file 5 — Table S3. Univariate and multivariate analyses of factors associated with survival of 90 GC patients. (DOCX 19 kb) [file 13046_2018_729_MOESM5_ESM.docx]

**Additional file 5: Table S3.** Univariate and multivariate analyses of factors associated with survival of 90 GC patients

| Variables | Univariate analysis | | |  | Multivariate analysis | | |
| --- | --- | --- | --- | --- | --- | --- | --- |
|  | HR | 95%CI | *P* |  | HR | 95%CI | *P* |
| Age (years) | 1.224 | 0.703-2.130 | 0.475 |  |  |  |  |
| Gender | 0.893 | 0.513-1.554 | 0.689 |  |  |  |  |
| Maximal tumor size (cm) | 0.974 | 0.075-1.623 | **0.028** |  | 0.608 | 0.329-1.123 | 0.112 |
| Pathological stage | 1.337 | 0.756-2.365 | 0.319 |  |  |  |  |
| Clinical stage | 0.245 | 0.178-0.973 | **<0.001** |  | 0.424 | 0.010-1.442 | **0.041** |
| T classification | 1.430 | 0.759-2.784 | 0.135 |  |  |  |  |
| Lymph node metastasis | 0.550 | 0.191-1.555 | **<0.001** |  | 0.239 | 0.072-0.796 | **0.020** |
| N classification | 0.391 | 0.098-1.176 | **<0.001** |  | 0.920 | 0.447-1.821 | 0.731 |
| Dock6 expression | 0.307 | 0.198-0.962 | **0.001** |  | 0.401 | 0.180-0.893 | **0.025** |
