# Supplementary material for: miR-148b-3p inhibits gastric cancer metastasis by inhibiting the Dock6/Rac1/Cdc42 axis
Source: J Exp Clin Cancer Res. 2018 Mar 27;37:71. doi: 10.1186/s13046-018-0729-z (PMC5872400; doi:10.1186/s13046-018-0729-z)
Supplement: Supplementary file 7 — Table S4. microRNAs that could target Dock6 as predicted by Target scan, Microcosm, miRDB and PicTar databases. (DOCX 22 kb) [file 13046_2018_729_MOESM7_ESM.docx]

**Additional file 7: Table S4. microRNAs that could target Dock6**

| **miRNAs** | **Gene symbols** | **TargetScan** | **MicroCosm** | **PicTar** | **miRDB** |
| --- | --- | --- | --- | --- | --- |
| hsa-miR-148b-3p | DOCK6 | 1 | 1 | 1 | 1 |
| hsa-miR-486-3p | DOCK6 | 1 | 1 | 0 | 1 |
| hsa-miR-593-5p | DOCK6 | 1 | 1 | 0 | 0 |
| hsa-miR-644a | DOCK6 | 1 | 1 | 0 | 0 |
| hsa-miR-22-5p | DOCK6 | 1 | 1 | 0 | 0 |
| hsa-miR-218-1-3p | DOCK6 | 1 | 1 | 0 | 0 |
| hsa-miR-194-3p | DOCK6 | 1 | 1 | 0 | 0 |
| hsa-miR-4739 | DOCK6 | 1 | 0 | 0 | 1 |
| hsa-miR-548ac | DOCK6 | 1 | 0 | 0 | 1 |
| hsa-miR-7106-5p | DOCK6 | 1 | 0 | 0 | 1 |
| hsa-miR-548t-3p | DOCK6 | 1 | 0 | 0 | 0 |
| hsa-miR-4747-3p | DOCK6 | 1 | 0 | 0 | 0 |
| hsa-miR-5094 | DOCK6 | 1 | 0 | 0 | 0 |
| hsa-miR-367-3p | DOCK6 | 1 | 0 | 0 | 0 |
| hsa-miR-4717-3p | DOCK6 | 1 | 0 | 0 | 0 |
| hsa-miR-5685 | DOCK6 | 1 | 0 | 0 | 0 |
| hsa-miR-6795-3p | DOCK6 | 1 | 0 | 0 | 0 |
| hsa-miR-221-5p | DOCK6 | 1 | 0 | 0 | 0 |
| hsa-miR-219b-5p | DOCK6 | 1 | 0 | 0 | 0 |
| hsa-miR-8064 | DOCK6 | 1 | 0 | 0 | 0 |
| hsa-miR-4771 | DOCK6 | 1 | 0 | 0 | 0 |
| hsa-miR-2467-3p | DOCK6 | 1 | 0 | 0 | 0 |
| hsa-miR-6132 | DOCK6 | 1 | 0 | 0 | 0 |
| hsa-miR-3665 | DOCK6 | 1 | 0 | 0 | 0 |
| hsa-miR-6811-3p | DOCK6 | 1 | 0 | 0 | 0 |
| hsa-miR-5581-5p | DOCK6 | 1 | 0 | 0 | 0 |
| hsa-miR-133a-5p | DOCK6 | 1 | 0 | 0 | 0 |
| hsa-miR-138-5p | DOCK6 | 1 | 0 | 0 | 0 |
| hsa-miR-6828-5p | DOCK6 | 1 | 0 | 0 | 0 |
| hsa-miR-4740-5p | DOCK6 | 1 | 0 | 0 | 0 |
| hsa-miR-4515 | DOCK6 | 1 | 0 | 0 | 0 |
| hsa-miR-766-5p | DOCK6 | 1 | 0 | 0 | 0 |
| hsa-miR-3144-5p | DOCK6 | 1 | 0 | 0 | 0 |
| hsa-miR-4652-5p | DOCK6 | 1 | 0 | 0 | 0 |
| hsa-miR-567 | DOCK6 | 1 | 0 | 0 | 0 |
| hsa-miR-3691-5p | DOCK6 | 1 | 0 | 0 | 0 |
| hsa-miR-6501-5p | DOCK6 | 1 | 0 | 0 | 0 |
| hsa-miR-4423-5p | DOCK6 | 1 | 0 | 0 | 0 |
| hsa-miR-7111-3p | DOCK6 | 1 | 0 | 0 | 0 |
| hsa-miR-6881-3p | DOCK6 | 1 | 0 | 0 | 0 |
| hsa-miR-6810-5p | DOCK6 | 1 | 0 | 0 | 0 |
| hsa-miR-5587-5p | DOCK6 | 1 | 0 | 0 | 0 |
| hsa-miR-4774-3p | DOCK6 | 1 | 0 | 0 | 0 |
| hsa-miR-3152-5p | DOCK6 | 1 | 0 | 0 | 0 |
| hsa-miR-548ae | DOCK6 | 1 | 0 | 0 | 0 |
| hsa-miR-6768-5p | DOCK6 | 1 | 0 | 0 | 0 |
| hsa-miR-942-3p | DOCK6 | 1 | 0 | 0 | 0 |
| hsa-miR-6753-5p | DOCK6 | 1 | 0 | 0 | 0 |
| hsa-miR-28-3p | DOCK6 | 1 | 0 | 0 | 0 |
| hsa-miR-4773 | DOCK6 | 1 | 0 | 0 | 0 |
| hsa-miR-4323 | DOCK6 | 1 | 0 | 0 | 0 |
| hsa-miR-6735-5p | DOCK6 | 1 | 0 | 0 | 0 |
| hsa-miR-7160-3p | DOCK6 | 1 | 0 | 0 | 0 |
| hsa-miR-6890-5p | DOCK6 | 1 | 0 | 0 | 0 |
| hsa-miR-4733-3p | DOCK6 | 1 | 0 | 0 | 0 |
| hsa-miR-1324 | DOCK6 | 1 | 0 | 0 | 0 |
| hsa-miR-4302 | DOCK6 | 1 | 0 | 0 | 0 |
| hsa-miR-4685-5p | DOCK6 | 1 | 0 | 0 | 0 |
| hsa-miR-4292 | DOCK6 | 1 | 0 | 0 | 0 |
| hsa-miR-6734-3p | DOCK6 | 1 | 0 | 0 | 0 |
| hsa-miR-1909-3p | DOCK6 | 1 | 0 | 0 | 0 |
| hsa-miR-1538 | DOCK6 | 1 | 0 | 0 | 0 |
| hsa-miR-376b-5p | DOCK6 | 1 | 0 | 0 | 0 |
| hsa-miR-8056 | DOCK6 | 1 | 0 | 0 | 0 |
| hsa-miR-6826-3p | DOCK6 | 1 | 0 | 0 | 0 |
| hsa-miR-6780a-3p | DOCK6 | 1 | 0 | 0 | 0 |
| hsa-miR-6511b-5p | DOCK6 | 1 | 0 | 0 | 0 |
| hsa-miR-6514-3p | DOCK6 | 1 | 0 | 0 | 0 |
| hsa-miR-1269b | DOCK6 | 1 | 0 | 0 | 0 |
| hsa-miR-744-3p | DOCK6 | 1 | 0 | 0 | 0 |
| hsa-miR-129-5p | DOCK6 | 1 | 0 | 0 | 0 |
| hsa-miR-6769a-3p | DOCK6 | 1 | 0 | 0 | 0 |
| hsa-miR-1322 | DOCK6 | 1 | 0 | 0 | 0 |
| hsa-miR-500b-3p | DOCK6 | 1 | 0 | 0 | 0 |
| hsa-miR-4269 | DOCK6 | 1 | 0 | 0 | 0 |
| hsa-miR-3607-5p | DOCK6 | 1 | 0 | 0 | 0 |
| hsa-miR-1180-5p | DOCK6 | 1 | 0 | 0 | 0 |
| hsa-miR-6089 | DOCK6 | 1 | 0 | 0 | 0 |
| hsa-miR-657 | DOCK6 | 1 | 0 | 0 | 0 |
| hsa-miR-6131 | DOCK6 | 1 | 0 | 0 | 0 |
| hsa-miR-2116-5p | DOCK6 | 1 | 0 | 0 | 0 |
| hsa-miR-6808-3p | DOCK6 | 1 | 0 | 0 | 0 |
| hsa-miR-147a | DOCK6 | 1 | 0 | 0 | 0 |
| hsa-miR-5683 | DOCK6 | 1 | 0 | 0 | 0 |
| hsa-miR-6856-3p | DOCK6 | 1 | 0 | 0 | 0 |
| hsa-miR-3130-5p | DOCK6 | 1 | 0 | 0 | 0 |
| hsa-miR-6757-5p | DOCK6 | 1 | 0 | 0 | 0 |
| hsa-miR-4688 | DOCK6 | 1 | 0 | 0 | 0 |
| hsa-miR-3163 | DOCK6 | 1 | 0 | 0 | 0 |
| hsa-miR-3675-5p | DOCK6 | 1 | 0 | 0 | 0 |
| hsa-miR-6834-3p | DOCK6 | 1 | 0 | 0 | 0 |
| hsa-miR-4501 | DOCK6 | 1 | 0 | 0 | 0 |
| hsa-miR-4457 | DOCK6 | 1 | 0 | 0 | 0 |
| hsa-miR-6736-3p | DOCK6 | 1 | 0 | 0 | 0 |
| hsa-miR-6887-3p | DOCK6 | 1 | 0 | 0 | 0 |
| hsa-miR-3162-3p | DOCK6 | 1 | 0 | 0 | 0 |
| hsa-miR-6803-3p | DOCK6 | 1 | 0 | 0 | 0 |
| hsa-miR-4308 | DOCK6 | 1 | 0 | 0 | 0 |
| hsa-miR-4649-3p | DOCK6 | 1 | 0 | 0 | 0 |
| hsa-miR-4677-3p | DOCK6 | 1 | 0 | 0 | 0 |
| hsa-miR-5008-5p | DOCK6 | 1 | 0 | 0 | 0 |
| hsa-miR-3682-3p | DOCK6 | 1 | 0 | 0 | 0 |
| hsa-miR-4711-5p | DOCK6 | 1 | 0 | 0 | 0 |
| hsa-miR-5699-5p | DOCK6 | 1 | 0 | 0 | 0 |
| hsa-miR-214-5p | DOCK6 | 1 | 0 | 0 | 0 |
| hsa-miR-7160-5p | DOCK6 | 1 | 0 | 0 | 0 |
| hsa-miR-1288-3p | DOCK6 | 1 | 0 | 0 | 0 |
| hsa-miR-4791 | DOCK6 | 1 | 0 | 0 | 0 |
| hsa-miR-4764-5p | DOCK6 | 1 | 0 | 0 | 0 |
| hsa-miR-34a-5p | DOCK6 | 1 | 0 | 0 | 0 |
| hsa-miR-6721-5p | DOCK6 | 1 | 0 | 0 | 0 |
| hsa-miR-3191-3p | DOCK6 | 1 | 0 | 0 | 0 |
| hsa-miR-6879-3p | DOCK6 | 1 | 0 | 0 | 0 |
| hsa-miR-4504 | DOCK6 | 1 | 0 | 0 | 0 |
| hsa-miR-542-3p | DOCK6 | 1 | 0 | 0 | 0 |
| hsa-miR-7973 | DOCK6 | 1 | 0 | 0 | 0 |
| hsa-miR-3657 | DOCK6 | 1 | 0 | 0 | 0 |
| hsa-miR-3911 | DOCK6 | 1 | 0 | 0 | 0 |
| hsa-miR-6867-5p | DOCK6 | 1 | 0 | 0 | 0 |
| hsa-miR-4326 | DOCK6 | 1 | 0 | 0 | 0 |
| hsa-miR-6755-3p | DOCK6 | 1 | 0 | 0 | 0 |
| hsa-miR-340-5p | DOCK6 | 1 | 0 | 0 | 0 |
| hsa-miR-137 | DOCK6 | 1 | 0 | 0 | 0 |
| hsa-miR-197-3p | DOCK6 | 1 | 0 | 0 | 0 |
| hsa-miR-6802-3p | DOCK6 | 1 | 0 | 0 | 0 |
| hsa-miR-6883-3p | DOCK6 | 1 | 0 | 0 | 0 |
| hsa-miR-6894-3p | DOCK6 | 1 | 0 | 0 | 0 |
| hsa-miR-4752 | DOCK6 | 1 | 0 | 0 | 0 |
| hsa-miR-6818-5p | DOCK6 | 1 | 0 | 0 | 0 |
| hsa-miR-4677-5p | DOCK6 | 1 | 0 | 0 | 0 |
| hsa-miR-5010-3p | DOCK6 | 1 | 0 | 0 | 0 |
| hsa-miR-148a-3p | DOCK6 | 0 | 1 | 1 | 1 |
| hsa-miR-152-3p | DOCK6 | 0 | 1 | 1 | 1 |
| hsa-miR-548d-3p | DOCK6 | 0 | 1 | 0 | 1 |
| hsa-miR-451a | DOCK6 | 0 | 1 | 0 | 0 |
| hsa-miR-606 | DOCK6 | 0 | 1 | 0 | 0 |
| hsa-miR-944 | DOCK6 | 0 | 1 | 0 | 0 |
| hsa-miR-617 | DOCK6 | 0 | 1 | 0 | 0 |
| hsa-miR-564 | DOCK6 | 0 | 1 | 0 | 0 |
| hsa-miR-34c-5p | DOCK6 | 0 | 1 | 0 | 0 |
| hsa-miR-409-5p | DOCK6 | 0 | 1 | 0 | 0 |
| hsa-miR-583 | DOCK6 | 0 | 1 | 0 | 0 |
| hsa-miR-941 | DOCK6 | 0 | 1 | 0 | 0 |
| hsa-miR-218-2-3p | DOCK6 | 0 | 1 | 0 | 0 |
| hsa-miR-331-5p | DOCK6 | 0 | 1 | 0 | 0 |
| hsa-miR-188-3p | DOCK6 | 0 | 1 | 0 | 0 |
| hsa-miR-29b-2-5p | DOCK6 | 0 | 1 | 0 | 0 |
| hsa-miR-602 | DOCK6 | 0 | 1 | 0 | 0 |
| hsa-miR-455-3p | DOCK6 | 0 | 1 | 0 | 0 |
| hsa-miR-29b-1-5p | DOCK6 | 0 | 1 | 0 | 0 |
| hsa-miR-376a-5p | DOCK6 | 0 | 1 | 0 | 0 |
| hsa-miR-183-3p | DOCK6 | 0 | 1 | 0 | 0 |
| hsa-miR-195-5p | DOCK6 | 0 | 1 | 0 | 0 |
| hsa-miR-21-5p | DOCK6 | 0 | 1 | 0 | 0 |
| hsa-miR-139-5p | DOCK6 | 0 | 1 | 0 | 0 |
| hsa-miR-330-5p | DOCK6 | 0 | 1 | 0 | 0 |
| hsa-miR-423-5p | DOCK6 | 0 | 1 | 0 | 0 |
| hsa-miR-18b-3p | DOCK6 | 0 | 1 | 0 | 0 |
| hsa-miR-24-3p | DOCK6 | 0 | 1 | 0 | 0 |
| hsa-miR-379-5p | DOCK6 | 0 | 1 | 0 | 0 |
| hsa-miR-541-3p | DOCK6 | 0 | 1 | 0 | 0 |
| hsa-miR-654-5p | DOCK6 | 0 | 1 | 0 | 0 |
| hsa-miR-668-3p | DOCK6 | 0 | 1 | 0 | 0 |
| hsa-miR-933 | DOCK6 | 0 | 1 | 0 | 0 |
| hsa-miR-133b | DOCK6 | 0 | 1 | 0 | 0 |
| hsa-miR-133a-3p | DOCK6 | 0 | 1 | 0 | 0 |
| hsa-miR-548h-3p | DOCK6 | 0 | 0 | 0 | 1 |
| hsa-miR-4756-5p | DOCK6 | 0 | 0 | 0 | 1 |
| hsa-miR-1321 | DOCK6 | 0 | 0 | 0 | 1 |
| hsa-miR-301a-3p | DOCK6 | 0 | 0 | 1 | 0 |
| hsa-miR-130b-3p | DOCK6 | 0 | 0 | 1 | 0 |
| hsa-miR-130a-3p | DOCK6 | 0 | 0 | 1 | 0 |
