# Supplementary material for: miR-148b-3p inhibits gastric cancer metastasis by inhibiting the Dock6/Rac1/Cdc42 axis
Source: J Exp Clin Cancer Res. 2018 Mar 27;37:71. doi: 10.1186/s13046-018-0729-z (PMC5872400; doi:10.1186/s13046-018-0729-z)
Supplement: Supplementary file 8 — Table S5. Correlation between miR-148b-3p expression and pathological characteristics of GC patients. (DOCX 19 kb) [file 13046_2018_729_MOESM8_ESM.docx]

**Additional file 8: Table S5.** Correlation between miR-148b-3p expression and pathological characteristics of GC patients

| Variables |  | No. | miR-148b-3p (n=90)  -(n=49) +(n=41) | | *P* value |
| --- | --- | --- | --- | --- | --- |
| Age | ≤60 | 32 | 20 | 12 | 0.254 |
|  | >60 | 58 | 29 | 29 |  |
| Gender | Male | 62 | 35 | 27 | 0.569 |
|  | Female | 28 | 14 | 14 |  |
| Max tumor size | ≤5cm | 30 | 16 | 14 | 0.881 |
|  | >5cm | 60 | 33 | 27 |  |
| Differentiation | Well /Moderate | 34 | 19 | 15 | 0.831 |
|  | Poor | 56 | 30 | 26 |  |
| T Classification | T1-T2 | 11 | 5 | 6 | 0.523 |
|  | T3-T4 | 79 | 44 | 35 |  |
| Lymph node metastasis | - | 25 | 8 | 17 | **0.008** |
|  | + | 65 | 41 | 24 |  |
| N classification | N0-N1 | 35 | 13 | 22 | **0.009** |
|  | N2-N3 | 55 | 36 | 19 |  |
| Clinical stage | I-II | 35 | 14 | 21 | **0.028** |
|  | III-IV | 55 | 35 | 20 |  |
